# Sustained Control of Serpiginous Choroiditis with the Fluocinolone Acetonide 0.18 mg Intravitreal Implant

**DOI:** 10.1155/2022/3962221

**Published:** 2022-12-20

**Authors:** Yousuf Siddiqui, Olufemi E. Adams, Michael A. Simmons, Justin Yamanuha, Dara D. Koozekanani

**Affiliations:** ^1^University of Minnesota Medical School, University of Minnesota, 420 Delaware St SE, Minneapolis, MN 55455, USA; ^2^Department of Ophthalmology and Visual Neurosciences, University of Minnesota, 516 Delaware Street SE, Minneapolis, MN 55455, USA

## Abstract

**Purpose:**

To describe an alternative treatment for a patient with serpiginous choroiditis (SC) who was not tolerant to systemic therapies.

**Methods:**

Case report of a patient with serpiginous choroiditis with their clinical course followed with ophthalmic examinations and multimodal imaging overtime. *Patients and Results*. A 57-year-old female with serpiginous choroiditis was treated for seven years with numerous therapies including systemic steroids, immunosuppressive agents, and repeated dexamethasone intravitreal implants. The patient was intolerant of systemic therapies and would flare if dexamethasone injections were performed less frequently than every 8 weeks, making a viable long-term treatment plan problematic. Following one injection of the fluocinolone acetonide 0.18 mg intravitreal implant, she has experienced sustained control for 20 months. *Discussion and Conclusions*. Real-world treatment of SC is complex as long-term control is necessary, and associated side effects of the therapies provided may limit sustained use. The fluocinolone acetonide implant lasts 36 months and may be an alternative long-term management option, especially in the setting of systemic medication intolerance for some patients with SC.

## 1. Introduction

Serpiginous Choroiditis (SC) is a rare inflammatory condition that usually occurs bilaterally and is a cyclically progressive disease characterized by periods of dormancy requiring continued treatment to control inflammation and prevent severe vision loss from complications such as macular scarring and choroidal neovascular membranes [1]. Management of SC usually requires ongoing systemic immunomodulatory agents [2, 3]; however, these can have serious adverse systemic effects.

YUTIQ (EyePoint Pharmaceuticals, Inc., MA, USA) is a sustained-release, 0.18 mg fluocinolone acetonide intravitreal implant that has demonstrated success in treating noninfectious uveitis [4, 5]. To our knowledge, no data exists in the literature on the efficacy of the fluocinolone acetonide intravitreal implant in managing SC. Herein, we present a case of SC that has now been controlled for 20 months following a single injection of the fluocinolone acetonide intravitreal implant in one eye.

## 2. Case Report

This is a case report of a 57-year-old female who presented to our institution with serpiginous choroiditis in both eyes (worse in the left than the right). She had originally been diagnosed 20 years prior, with prior treatment including oral prednisone and subtenon's triamcinolone acetonide injections. She presented with a visual acuity of 20/70 right eye (OD) and hand-motion left eye (OS). Dilated funduscopic examination was remarkable for chorioretinal scars in the posterior pole with an active area of fovea-threatening choroiditis in the right eye. The left eye had extensive chorioretinal scars in the posterior pole contiguous with the optic nerve head and a fibrotic subfoveal scar with subretinal fluid and blood.

**Figure 1:**
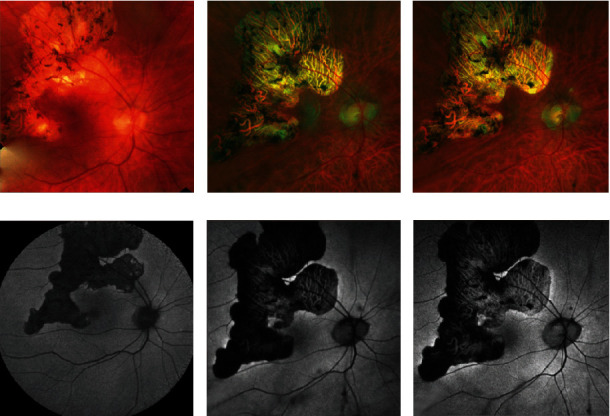
Fundus image and fundus autofluorescence of right eye on initial presentation (a, d), right eye 6 years later—prior to fluocinolone acetonide intravitreal implant—(b, e), and right eye 15 months after fluocinolone acetonide intravitreal implant (c, f).

**Figure 2:**
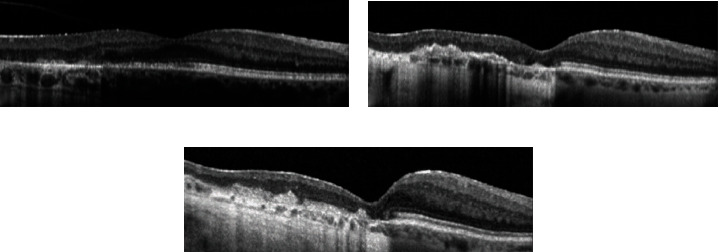
Optical coherence tomography (OCT) of the right eye on initial presentation (a), right eye 6 years later—prior to fluocinolone acetonide intravitreal implant—(b), and right eye 15 months after fluocinolone acetonide intravitreal implant (c).

Fundus autofluorescence (FAF) depicted hypofluorescent serpentine-like lesions, with a hyperautofluorescent edge in close proximity to the fovea OD (Figure 1). Optical coherence tomography (OCT) revealed temporal outer retinal atrophic changes without fluid OD (Figure 2). On fluorescein angiography, these lesions exhibited early hypofluorescence with late staining at the edges in both eyes. Laboratory testing for tuberculosis, syphilis, sarcoidosis, and toxoplasmosis were negative.

The SC was initially treated with a combination of oral prednisone, azathioprine, and cyclosporine. Despite treatment, the inflammation persisted, and after three months, immunosuppressive therapy was escalated to include a 3-month course of intravenous cyclophosphamide and intravitreal triamcinolone acetonide (IVTA). The patient was unable to tolerate side effects from this regimen and thus was changed to mycophenolate with oral prednisone after another 3 months. She was unable to tolerate this either, and ultimately stopped further treatment nine months after her initial presentation. She also developed a juxtafoveal choroidal neovascular membrane (CNVM) requiring intravitreal bevacizumab (IVB) twice. The SC remained in remission for two years without treatment other than a preventative IVTA and oral steroid burst given at the time of cataract surgery with intraocular lens implantation during this time period. She also required occasional IVB for the CNVM.

After 2 years, she returned with vision complaints from a reactivation of the SC and CNVM. Her visual acuity was still 20/60 in her right eye. At this point, she resumed systemic therapy and initiated a new course of cyclophosphamide and oral prednisone. She also received IVB for her CNVM. However after 3 months, she again stopped further systemic treatment due to intolerable side effects. At that time, visual acuity was reduced to 20/200 in the right eye.

Within 4 months of stopping treatment, she had recurrent inflammation again. To avoid further systemic side effects, a local steroid treatment regimen was initiated with repeated IVTA that was then transitioned to dexamethasone 0.7 mg intravitreal implants (Ozurdex, Allergan) on a pro re nata (PRN) basis. She also received ranibizumab PRN for the CNVM. She repeatedly required dexamethasone implant injections every 2-4 months to treat recurrent inflammation; thus, the regimen was changed to scheduled dexamethasone implant injections every 8 weeks. During the following 3 years, after receiving 4 ranibizumab intravitreal injections and 14 dexamethasone implant injections, her visual acuity was 20/400 and intraocular pressure was stable with anti-ocular hypertensive drops.

In an effort to reduce the treatment burden from frequent injections, an attempt was made to reinitiate mycophenolate, but she was intolerant to therapy within 2 months. Fluocinolone acetonide intravitreal implant was then provided to the right eye. This was placed 6 weeks after the last dexamethasone implant, and since then, no further intravitreal or systemic treatments have been required. The patient has undergone regular monitoring with clinical exams and multimodal imaging, including OCT and FAF. Upon evaluation 15 months after fluocinolone acetonide implant placement, there was no recurrence of inflammation evident (Figures 1 and 2), and repeat assessment at 20 months revealed stability of findings. The visual acuity in the right eye remained stable at 20/300. Her intraocular pressure has been stable without the need for additional therapy. Through this entire course, her left eye has been stable at a visual acuity of hand motion and no additional therapies have been performed.

## 3. Discussion

The gold standard of treatment for SC is the use of immunosuppressants in combination with steroids, as this has been shown to shorten the duration of acute attacks and prevent recurrences [6]. In our case we highlight the real-world experience of the complex treatment course of a patient with SC. Over the course of seven years, our patient tried multiple systemic immunosuppressive therapies to control the SC. She was intolerant to all the systemic therapies she tried, however, and experienced several recurrences which led to disease progression and vision loss.

She was ultimately able to achieve disease stability with local treatment using scheduled dexamethasone intravitreal implants every 8 weeks. However, this created a significant treatment burden for her. With the introduction of the fluocinolone acetonide intravitreal implant, the patient experienced rapid stabilization of her vision and has remained quiescent for fifteen months without the need for additional systemic or local treatment and without any significant adverse effects. We propose that the fluocinolone acetonide 0.18 mg intravitreal implant may be considered as an alternative long-term management option for serpiginous choroiditis for some patients, especially those unable to tolerate systemic medications.

## Data Availability

Data is available on request by contacting the corresponding author: Olufemi E. Adams, University of Minnesota, 516 Delaware St. SE., Minneapolis, MN 55455, tel.: 612-626-2452, fax: 612-904-4355, email: adams977@umn.edu.

## Additional Points

*Summary Statement.* Serpiginous choroiditis is a progressive, chronic inflammatory uveitic condition which often requires systemic immunomodulatory agents. We present a case of a 57-year-old female with serpiginous choroiditis intolerant of systemic therapies during seven years of treatment, who has achieved quiescence and sustained control for 20 months after one fluocinolone acetonide intravitreal implant.

## References

[1] Lim W. K., Buggage R. R., Nussenblatt R. B. (2005). Serpiginous choroiditis. *Survey of Ophthalmology*.

[2] Samy A., Lightman S., Ismetova F., Talat L., Tomkins-Netzer O. (2014). Role of autofluorescence in inflammatory/infective diseases of the retina and choroid. *Journal of Ophthalmology*.

[3] Akpek E. K., Jabs D. A., Tessler H. H., Joondeph B. C., Foster C. S. (2002). Successful treatment of serpiginous choroiditis with alkylating agents. *Ophthalmology*.

[4] Pavesio C., Heinz C. (2022). Non-infectious uveitis affecting the posterior segment treated with fluocinolone acetonide intravitreal implant: 3-year fellow eye analysis. *Eye*.

[5] Jaffe G. J., Pavesio C. E. (2020). Effect of a fluocinolone acetonide insert on recurrence rates in noninfectious intermediate, posterior, or panuveitis: three-year results. *Ophthalmology*.

[6] Vianna R. N., Ozdal P. C., Deschênes J., Burnier M. N. (2006). Combination of azathioprine and corticosteroids in the treatment of serpiginous choroiditis. *Canadian Journal of Ophthalmology*.

